# Supplementary material for: Conditional chemoconnectomics (cCCTomics) as a strategy for efficient and conditional targeting of chemical transmission
Source: eLife. 2024 Apr 30;12:RP91927. doi: 10.7554/eLife.91927 (PMC11060718; doi:10.7554/eLife.91927)
Supplement: Figure 3—source data 3. [file elife-91927-fig3-data3.zip › Figure 3 source data3 part 1.zip!/Figure 3-Source data 3-part1/1071f2-1-CRISPResso_Report_ztwktvfp.zip!/CRISPResso_on_1071f2_4/3b.Insertion_deletion_substitutions_size_hist.pdf]

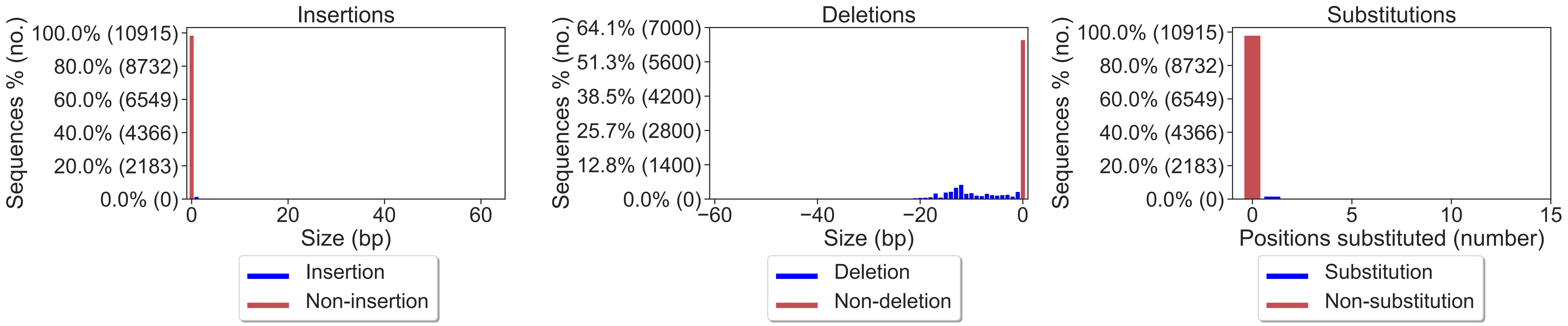
Insertions
Sequences % (no.)
100.0% (10915)
80.0% (8732)
60.0% (6549)
40.0% (4366)
20.0% (2183)
0.0% (0)
0
20
40
60
Size (bp)
Insertion
Non-insertion
Deletions
Sequences % (no.)
64.1% (7000)
51.3% (5600)
38.5% (4200)
25.7% (2800)
12.8% (1400)
0.0% (0)
−60
−40
−20
0
Size (bp)
Deletion
Non-deletion
Substitutions
Sequences % (no.)
100.0% (10915)
80.0% (8732)
60.0% (6549)
40.0% (4366)
20.0% (2183)
0.0% (0)
0
5
10
15
Positions substituted (number)
Substitution
Non-substitution
